# Novel insights into phage biology of the pathogen *Clostridioides difficile* based on the active virome

**DOI:** 10.3389/fmicb.2024.1374708

**Published:** 2024-03-21

**Authors:** Miriam A. Schüler, Rolf Daniel, Anja Poehlein

**Affiliations:** Genomic and Applied Microbiology and Göttingen Genomics Laboratory, Institute of Microbiology and Genetics, Georg-August-University Göttingen, Göttingen, Germany

**Keywords:** *Clostridioides difficile*, temperate phage, phage induction, secondary bile salt, deoxycholate, mobile genetic element

## Abstract

The global pathogen *Clostridioides difficile* is a well-studied organism, and researchers work on unraveling its fundamental virulence mechanisms and biology. Prophages have been demonstrated to influence *C. difficile* toxin expression and contribute to the distribution of advantageous genes. All these underline the importance of prophages in *C. difficile* virulence. Although several *C. difficile* prophages were sequenced and characterized, investigations on the entire active virome of a strain are still missing. Phages were mainly isolated after mitomycin C-induction, which does not resemble a natural stressor for *C. difficile.* We examined active prophages from different *C. difficile* strains after cultivation in the absence of mitomycin C by sequencing and characterization of particle-protected DNA. Phage particles were collected after standard cultivation, or after cultivation in the presence of the secondary bile salt deoxycholate (DCA). DCA is a natural stressor for *C. difficile* and a potential prophage-inducing agent. We also investigated differences in prophage activity between clinical and non-clinical *C. difficile* strains. Our experiments demonstrated that spontaneous prophage release is common in *C. difficile* and that DCA presence induces prophages. Fourteen different, active phages were identified by this experimental procedure. We could not identify a definitive connection between clinical background and phage activity. However, one phage exhibited distinctively higher activity upon DCA induction in the clinical strain than in the corresponding non-clinical strain, although the phage is identical in both strains. We recorded that enveloped DNA mapped to genome regions with characteristics of mobile genetic elements other than prophages. This pointed to mechanisms of DNA mobility that are not well-studied in *C. difficile* so far. We also detected phage-mediated lateral transduction of bacterial DNA, which is the first described case in *C. difficile*. This study significantly contributes to our knowledge of prophage activity in *C. difficile* and reveals novel aspects of *C. difficile* (phage) biology.

## 1 Introduction

The pathogen *Clostridioides difficile* significantly contributes to nosocomial infections worldwide (Balsells et al., 2019). A *C. difficile* infection mainly establishes after antibiotic treatment due to diverse resistances in *C. difficile* strains combined with the disturbed intestinal microflora (Spigaglia, 2016). An intact microbiome usually provides resistance to *C. difficile* colonization and disease manifestation by producing different secondary bile salts such as lithocholate and deoxycholate (Sorg and Sonenshein, 2008). These compounds primarily aid the human intestines in digesting lipid nutrients due to their detergent character (Ridlon et al., 2006). However, they also impede *C. difficile* spore germination and cell growth (Thanissery et al., 2017). Symptoms of an established *C. difficile* infection are caused by its toxins, predominantly toxin A and B, which are encoded in the pathogenicity locus (Chandrasekaran and Borden Lacy, 2017). Symptom severity ranges from mild to severe manifestation, which can also include the death of the infected individual (Balsells et al., 2019). The personal health condition significantly affects resistance against a *C. difficile* infection, but the infecting strain is of relevance as well (Czepiel et al., 2019). Different *C. difficile* strains are linked to divergent virulence, and various aspects such as toxin production levels or secondary bile salt resistance were shown to correlate with disease severity (Lewis et al., 2017; Alonso et al., 2022). However, these studies also partially contradict the concluded relevance of specific features on virulence. In addition to general virulence factors, mobile genetic elements (MGEs) also contribute to *C. difficile* virulence (Govind et al., 2009; Sekulovic et al., 2011; Goh et al., 2013; Smits et al., 2022). Those elements play a key role in fast adaptation to environmental conditions via horizontal gene transfer (HGT) (la Cruz Fernando and Davies, 2000). *C. difficile* genomes harbor various MGEs, including prophages (Sebaihia et al., 2006). Prophages are widespread among the species *C. difficile*, and multiple prophages can exist within the same host (Fortier, 2018). Primarily, prophages were assumed to affect *C. difficile* virulence solely by encoding advantageous traits such as antibiotic resistance, which was demonstrated by phage-mediated transduction of an erythromycin resistance (Goh et al., 2013). Prophages were shown to influence toxin production in *C. difficile* strains by down- or upregulation of toxin gene expression via encoded transcriptional regulators (Govind et al., 2009; Sekulovic et al., 2011). Moreover, phages can influence the production of a protective biofilm or support the release of virulence-associated proteins and DNA (Fortier and Sekulovic, 2013; Nanda et al., 2015). These findings drew further attention to the influence of phages on *C. difficile* virulence. Phage research commonly works with prophage induction by introducing stressors such as UV radiation or mitomycin C. Meanwhile, studies confirmed spontaneous prophage release from *C. difficile* isolates, and clinically relevant antibiotics were also investigated for their phage-inducing effect (Meessen-Pinard et al., 2012; Nale et al., 2012). All experiments on *C. difficile* phages, however, worked with cultivation conditions that do not represent the actual habitat. Some components of the intestinal environment are stressful for *C. difficile*, such as the secondary bile salts. One of the prominent secondary bile salts is deoxycholate (DCA), which can promote biofilm formation in *C. difficile* (Dubois et al., 2019). DCA thereby supports bacterial persistence in the host and facilitates disease manifestation and relapse. A biofilm not only protects the bacterial cells from antimicrobial substances but also supports the adaptation of the bacterial population via HGT among the participating cells that are in close contact with the biofilm structure (Ghigo, 2001; Lécuyer et al., 2018). It was further demonstrated that DCA induces the bacterial SOS response (Kandell and Bernstein, 1991). Activation of the SOS response in turn induces prophages, leads to phage particle production, and releases via host cell lysis (Hu et al., 2021). It is therefore likely that DCA induces prophages as well, which would be a critical aspect in *C. difficile* biology and shed new light on genetic transfer *in vivo*.

In this study, we examined prophage activity in different *C. difficile* strains under spontaneous conditions and in the presence of DCA. The analyzed *C. difficile* strains were of non-clinical and clinical origin and corresponded pairwise to each other based on their sequence type (ST). Active prophage regions in these strains were identified by sequencing particle-protected DNA and were analyzed for DCA-induced activity. Possible differences between clinical and non-clinical strains, which could contribute to virulence, were also examined. The sequencing approach is more sensitive than electron microscopy or plaque assays, the commonly used detection methods for active *C. difficile* phages. We could therefore detect active prophages that might otherwise be missed due to insufficient activity but could contribute to HGT.

## 2 Methods

### 2.1 Strains and cultivation conditions

The *C. difficile* strains used in this study were of clinical or non-clinical background (personal communication), with four pairs of clinical and non-clinical strains corresponding to ST and one additional non-clinical strain (Table 1). Strains were routinely cultivated under anaerobic conditions in supplemented Brain Heart Infusion Broth (BHIS; supplemented with 0.5% yeast extract, 0.05% L-cysteine, 0.0001% Na-resazurin, purged with nitrogen) at 37°C. Putative prophage regions of the strains were predicted with PHASTEST (Wishart et al., 2023).

**Table 1:** *Clostridioides difficile* strains used in this study.

Strain	ST (Clade)	Toxin profile	Background	GenBank accession
TS3_3	1 (2)	A^+^B^+^CDT^+^	Non-clinical	CP134872
DSM 28196	1 (2)	A^+^B^+^CDT^+^	Clinical	CP012320
B1_2	3 (1)	A^+^B^+^CDT^─^	Non-clinical	CP132141-43
SC084-01-01	3 (1)	A^+^B^+^CDT^─^	Clinical	CP132146-48
J2_1	8 (1)	A^+^B^+^CDT^─^	Non-clinical	CP134690-1
SC083-01-01	8 (1)	A^+^B^+^CDT^─^	Clinical	CP132144-45
MA_1	11 (5)	A^+^B^+^CDT^+^	Non-clinical	CP132139-40
DSM 29747	11 (5)	A^+^B^+^CDT^+^	Clinical	CP019864
MA_2	340 (C-III)	A^─^B^─^CDT^─^	Non-clinical	CP129431-32

Strain information on ST (Clade), profile of toxins A, B, and CDT, clinical or non-clinical background, and GenBank accession of the genome is listed.

### 2.2 DCA tolerance assessment

The stress effect of DCA on the various strains was assessed via a minimum inhibitory concentration (MIC) assay and relative growth determinations at different concentrations. Cultivation was performed in cell culture plates (24 well for suspension cells, Sartorius AG, Göttingen, Germany) in an anaerobic tent (Coy Laboratory, Grass Lake, United States). Overnight cultures of *C. difficile* strains were cultivated as described above and used to inoculate 2 mL medium to a final OD_600_ of 0.05, with either BHIS medium alone or supplemented with DCA (sodium deoxycholate, Sigma-Aldrich Chemie Gmbh, Taufkirchen, Germany) in concentrations ranging from 0.255 mM to 1.2 mM, which cover the physiological concentration range in humans (Hamilton et al., 2007). Culture plates were incubated at 37°C for 22 h and kept anaerobic during the OD_600_ measurement in a Synergy 2 Plate Reader (Biotek Agilent Technologies Deutschland GmbH, Böblingen, Germany). Relative growth in the presence of DCA was calculated in relation to the untreated cultures. Each experiment was performed in triplicate.

Relative growth was visualized with ggplot2 (v3.4.2; Wickham, 2016) in RStudio (v2022.06.0; RStudio Team, 2020), and significance was determined with the Tukey’s ‘honest significant difference’ method implemented in the stats package (v4.2.0; Team, R Core, 2013).

### 2.3 Prophage induction and phage particle isolation

Phage particles were isolated from untreated and DCA-induced cultures. Two pre-warmed flasks of 55 mL BHIS for each isolate were inoculated 1:100 from an overnight culture and incubated at 37°C. Growth was monitored via OD_600_ measurements until an OD of ~0.6 (0.5–0.7). One culture for each isolate was induced with 0.255 mM (0.01%) DCA final concentration. The physiological concentration of DCA varies between individuals (Hamilton et al., 2007). We, therefore, used this concentration that is within the physiological range and was also used in various *C. difficile* studies regarding growth behavior or spore germination (Theriot et al., 2016; Lewis et al., 2017). The DCA solution was freshly prepared under anaerobic conditions, with DCA suspended in distilled water so that 100 μL inducing solution was required per 10 mL culture. The solution was sterilized by filtration (Filtropur S 0.2 μm, Sarstedt AG & Co. KG, Nümbrecht, Germany) and anaerobically added to the induction cultures. The second culture was not induced for analysis of spontaneous phage activity. Induced and non-induced cultures were further incubated until 22-h total incubation. The final OD_600_ of each culture was determined before isolating phage particles.

For phage particle isolation, the cells were pelleted via centrifugation at 4°C and 3,000 x *g* for 15 min. The remaining cells were removed by filtration of the supernatant with a 0.45-μm Filtropur S filter (Sarstedt). Phage particles were pelleted via centrifugation at 8°C and 20,000 x *g* for 1 h. The pellet was suspended in 1 mL SM buffer (50 mM Tris–HCl, 100 mM NaCl, 8 mM MgSO_4,_ 7 H_2_O, pH 7.4) supplemented with 0.5 mM CaCl_2_ (supporting phage stability and upcoming DNase treatment) and let soak overnight at 4°C. Particle suspension was further supported the next day by shaking at 150 rpm (LT-V Lab-Shaker, Adolf Kühner AG, Birsfelden, Germany) at room temperature for 2 h. The suspended samples were finally transferred to 2 mL DNA Low Binding microtubes (Sarstedt) for the following treatments using cut filter tips to reduce possible shearing. The samples were stored at 4°C.

### 2.4 Isolation of particle-protected DNA from phage samples

The phage DNA was isolated using the MasterPure Gram Positive DNA Purification Kit (Epicenter, Madison, WI, United States) with modifications. Prior to the phage DNA isolation, phage samples were supplemented with 2 μL of 100 mg/mL lysozyme solution (lysozyme from chicken egg white 177,000 U/mg; Serva, Heidelberg, Germany) suspended in SM buffer to remove remaining host cell debris and with 50 μg/mL final concentration RNase A (Biozym Scientific GmbH, Hess. Oldendorf, Germany) and 10 U Baseline-ZERO DNase (Biozym Scientific GmbH) to digest host nucleic acids. The samples were incubated at 37°C for 6 h with gentle inversion every 30 min. Fragments of host nucleic acids resulting from the digestion were removed by phage particle pelleting via centrifugation at 4°C and 20,000 x *g* for 1 h. The recovered pellet was suspended in 150 μL SM buffer. The suspension was supported by shaking at 150 rpm (LT-V Lab-Shaker) at room temperature for 10 min and slight flicking. EDTA (0.5 M, pH 8.0) was added to 10 mM final concentration for complete DNase inhibition.

Phage particles were lysed by adding 1% SDS (10% solution) and 2 μL Proteinase K (50 μg/μl; Biozym Scientific GmbH), incubation at 56°C for 1.5 h, and gentle inversion every 30 min. Subsequently, the samples were completely cooled down on ice before the addition of 130 μL MPC protein precipitation reagent (pre-cooled to −20°C). After mixing by gentle inversion, the proteins were pelleted via centrifugation at 4°C and 10,000 x *g* for 10 min. The DNA-containing supernatant was transferred to 1.5 mL DNA Low Binding microtubes (Sarstedt). The DNA was precipitated by addition of 0.3 M Na-acetate (3 M, pH 5.2), 10 mM MgCl_2_ (2 M), and 0.8 volume isopropanol at room temperature. The samples were inverted 40 times and incubated at room temperature for 10 min before centrifugation at 4°C and 15,000 x *g* for 30 min. The supernatant was removed carefully, and the DNA pellet was washed twice with 150 μL 70% ethanol (pre-cooled to −20°C) and centrifugation at 4°C and 15,000 x *g* for 5 to 10 min. The supernatant was removed, and the sample was briefly centrifuged again to collect all residual ethanol for final removal. DNA pellets were air-dried under a sterile bench and immediately suspended in 20 μL TE buffer. Complete DNA elution was supported by brief storage at 4°C and slight flicking, before final storage at −20°C. The DNA concentration was assessed with the Qubit 3.0 Fluorometer (Thermo Fisher Scientific, Waltham, MA, United States) using the HS dsDNA assay kit.

### 2.5 Phage DNA sequencing and sequencing read processing

The phage DNA was subjected to Illumina sequencing for dsDNA by paired-end library preparation with the Nextera XT DNA Library Preparation Kit (Illumina, San Diego, CA, United States) as recommended by the manufacturer. Libraries were sequenced using an Illumina MiSeq system and MiSeq Reagent Kit version 3 (2 × 300 bp, 600 cycles) as recommended by the manufacturer.

All following software was used in default mode. Sequencing raw reads were quality processed with fastp (v0.23.4; Chen et al., 2018) before removing the sequencing adapters with Trimmomatic (v0.39; Bolger et al., 2014). Processed reads were mapped onto the corresponding host genome using bowtie2 (v2.5.0), and the resulting SAM file was converted to a TDS file for bioinformatics analysis with the TraV software (Dietrich et al., 2014).

### 2.6 Data analysis of phage sequencing reads

TDS files of processed reads for the same isolate were together inspected in TraV (Dietrich et al., 2014) for read coverage, and reads were normalized by calculation of nucleotide activity per kilobase of exon model per million mapped reads (NPKM). This results in a value for each CDS corresponding to its read coverage in reference to the overall read amount. NPKM values were further normalized to account for the differing growth behavior under the induction conditions by transforming values to an OD_600_ of 2.0. In this way, NPKM values reflected phage abundance under the different conditions at the same cell density, which allows a better qualitative estimation of phage activity. OD normalization and visualization of NPKMs values were done in Rstudio (v2022.06.0; RStudio Team, 2020) using the packages tidyverse (v2.0.0; Wickham et al., 2019), ggforce (v0.4.1; Pedersen, 2022), and ggplot2 (v3.4.2; Wickham, 2016). NPKM values were plotted against the host genome with regard to the sequence start of the corresponding CDS, and original and normalized NPKM values were plotted together to visualize the effect of OD normalization. Phage regions predicted with PHASTEST (Wishart et al., 2023) were also implemented.

### 2.7 Phage genome annotation and gene content analysis

Active regions identified via sequence read mapping were extracted for a new genome annotation. Sequence ranges were thereby adopted from PHASTEST (Wishart et al., 2023) predictions or selected based on read mapping in TraV (Dietrich et al., 2014), if the predicted prophage region did not cover the entire mapped region. Annotation was customized for phage genomes using Pharokka (v1.3.2; Bouras et al., 2023) in default mode with sequence re-orientation to the large terminase subunit. If the large terminase subunit was not annotated automatically, it was determined via BLAST analysis (Johnson et al., 2008) and manually annotated. For specific genes and their encoded protein, additional analyses with protein BLAST (Johnson et al., 2008) and InterProScan (v5.63–95.0; Jones et al., 2014) were performed.

### 2.8 Phage genome-based classifications

Genome-based classifications were done with different bioinformatic analysis tools. An average nucleotide identity analysis (ANI) with pyani (v0.2.12; Pritchard, 2014) and MUMmer3 alignment (Kurtz et al., 2004) (ANIm) was used in default mode to compare the phages among each other. The assessment of the DNA-packaging strategy was performed based on the study of Rashid et al. (2016). The large terminase subunit was aligned at the protein sequence level with ClustalW, and a maximum-likelihood phylogenetic tree was constructed with the Whelan and Goldman (WAG) substitution model and otherwise default parameter with the software MEGA (v11.0.13; Tamura et al., 2021). The branches were collapsed in MEGA (Tamura et al., 2021) if none of our phages clustered within, and final modifications for visualization were done in Inkscape (v0.48).^1^ A nucleotide BLAST analysis (Johnson et al., 2008) with default parameters was performed to check for similarity to already known phage genomes and the prevalence in genomes of other *C. difficile* strains. BLAST results were ordered based on query coverage and hits with a query coverage below 10% were neglected unless relevant matches with higher coverages were not obtained. For assigning the phages to a morphological family of the order *Caudovirales*, phage genomes were inspected for the presence of baseplate proteins and sequence length of the tail length tape measure protein (Zinke et al., 2022). If the tail length tape measure protein was not annotated, it was identified via protein BLAST analysis (Johnson et al., 2008) and manually curated in the genome.

## 3 Results and discussion

### 3.1 DCA tolerance is linked to the genetic but not clinical strain background

**Figure 1:**
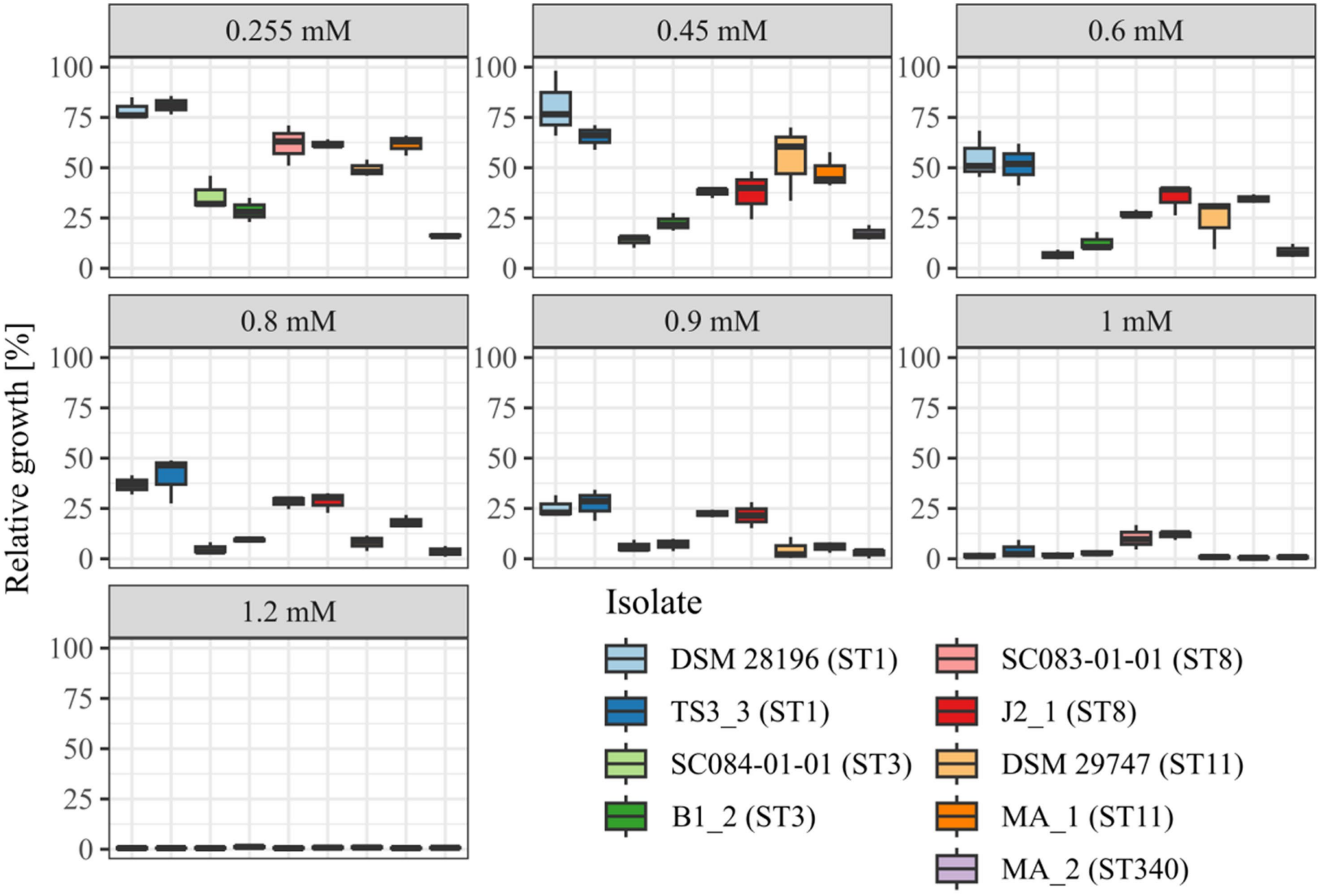
DCA tolerance at various concentrations. Tolerance of the strains to DCA was determined as relative growth compared to the reference culture (regarded as 100%) and MIC assay at concentrations from 0.255 mM to 1.2 mM. Strains of the same ST are depicted next to each other. No significant tolerance differences were observed within an ST at all concentrations.

Before investigating the effect of the secondary bile salt DCA on prophage activity in *C. difficile*, individual DCA tolerance of the various strains was assessed in the form of a MIC assay with relative growth determinations (Figure 1). DCA concentrations ranged from 0.255 mM to 1.2 mM, thereby comprising the physiological human concentration of DCA (Hamilton et al., 2007). All strains already exhibited reduced growth at the lowest concentration, which further decreased with increasing concentration. At all concentrations, strains of the same ST showed no significant difference in DCA tolerance, which implied comparable stress levels. Similar stress levels in turn might imply similar cellular strategies to cope with DCA-associated cellular damage but could also hint at similar DCA-induced prophage activity. In contrast, ST-specific tolerance differences were apparent across the DCA concentration range, with ST1 exhibiting the highest tolerance, followed by ST8, ST11, ST3, and, lastly, ST340, which was the most susceptible ST. Consequently, DCA tolerance correlated with the ST but not with the clinical background. The tolerance difference between the STs was most distinct at the lowest concentration, which was also used in the prophage induction experiments. Determined MICs ranged from 1 mM (ST11 and ST340) to 1.2 mM (ST1, ST3, ST8).

### 3.2 Sequencing-based assessments of prophage activity

Prophage prediction of all analyzed genomes exhibited various putative prophage regions, often with multiple incomplete and intact predicted regions in one genome (Supplementary Table S1). Active prophages were determined by sequencing of particle-protected DNA. Sequencing reads were mapped to the corresponding host genome. Normalized read coverage (NPKM values) represented phage abundance as a relative measure indicative of phage activity (Hertel et al., 2015). In the following, the term phage activity describes the production and resulting abundance of DNA-containing particles, and the mentioned NPKM values refer to the OD-normalized data. Sequencing of phage DNA libraries was successful for all samples except for strain DSM 29747, which was the only strain without a predicted intact prophage genomic region (Supplementary Table S1). This strain was therefore missing in further analyses.

**Figure 2:**
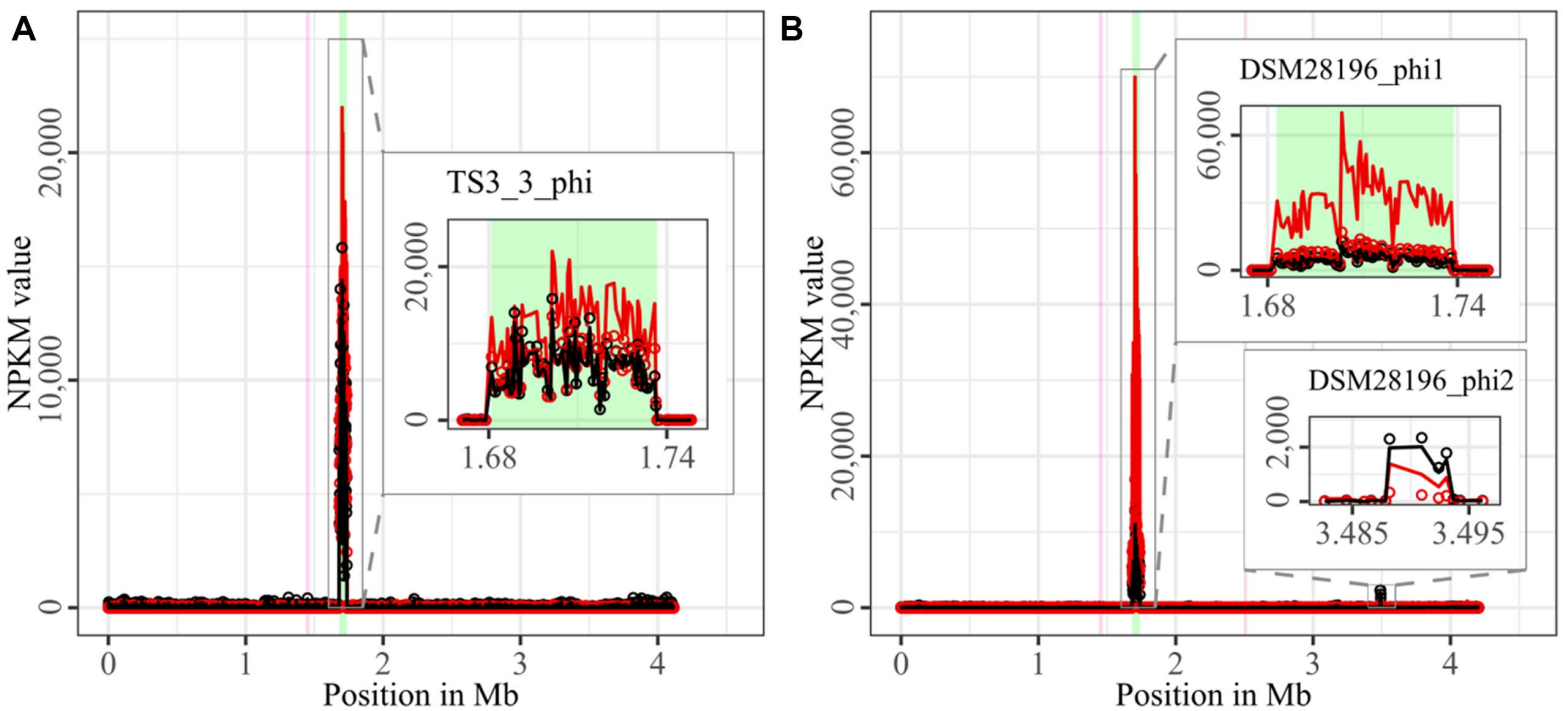
Coverage of phage sequencing reads of ST1-strains TS3_3 and DSM 28196. Sequencing reads of phage particles from spontaneous (black) and DCA-induced (red) release of strains **(A)** TS3_3 and **(B)** DSM 28196 were normalized to NPKM values (circles) with TraV (Dietrich et al., 2014), and NPKM values were additionally OD-normalized to OD_600_ = 2 (line graphs), before plotted against the chromosome (position in Mb) of the respective strain. Active regions were magnified for better visualization. Prophage regions predicted by PHASTEST (Wishart et al., 2023) are highlighted in the background (intact = green, incomplete = pink).

**Figure 3:**
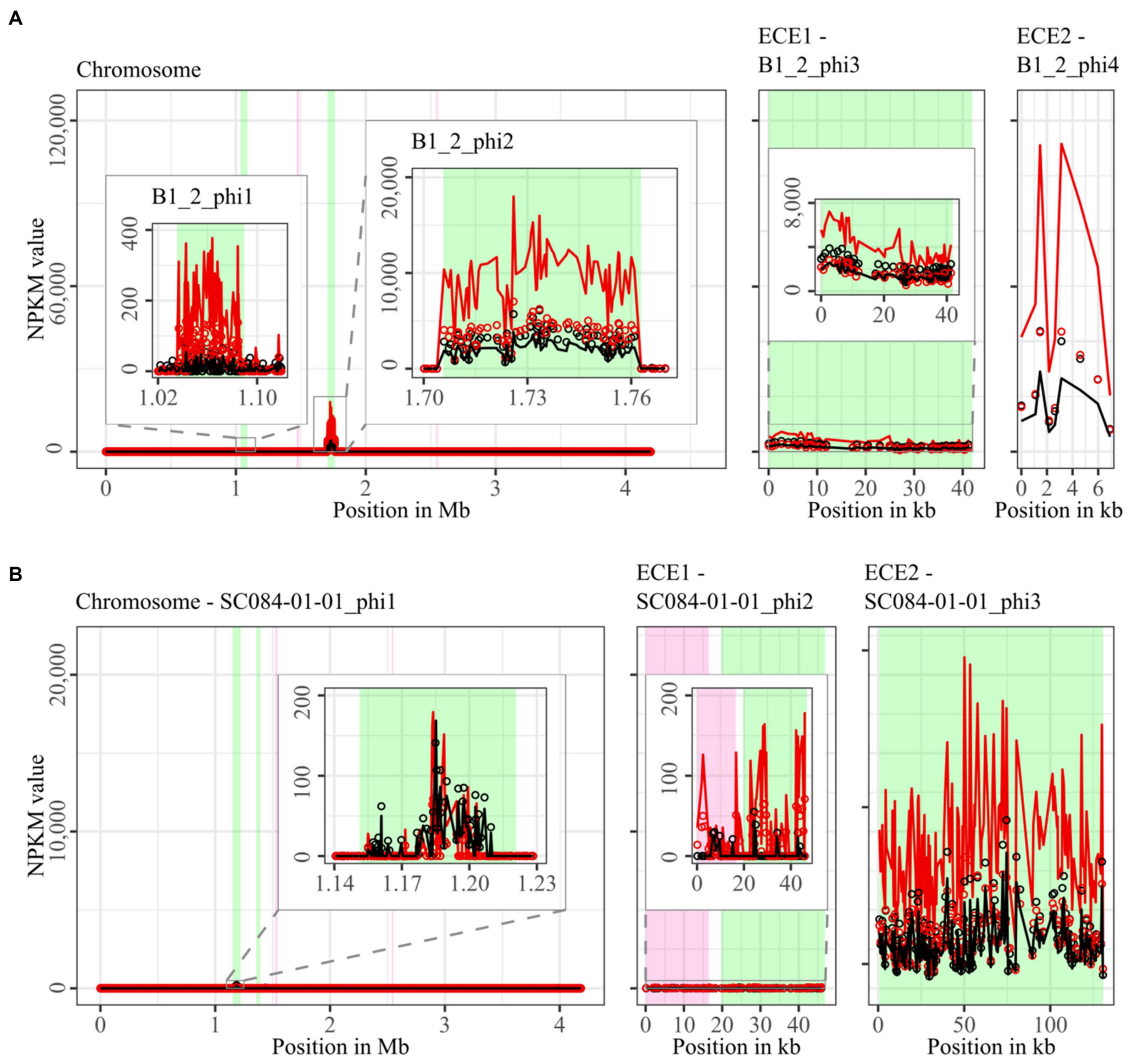
Coverage of phage sequencing reads of ST3-strains B1_2 and SC084-01-01. Sequencing reads of phage particles from spontaneous (black) and DCA-induced (red) release of strains **(A)** B1_2 and **(B)** SC084-01-01 were normalized to NPKM values (circles) with TraV (Dietrich et al., 2014), and NPKM values were additionally OD-normalized to OD_600_ = 2 (line graphs), before plotted against the chromosome (position in Mb) and ECE (position in kb) of the respective strain. Active regions were magnified for better visualization. Prophage regions predicted by PHASTEST (Wishart et al., 2023) are highlighted (intact = green, incomplete = pink).

**Figure 4:**
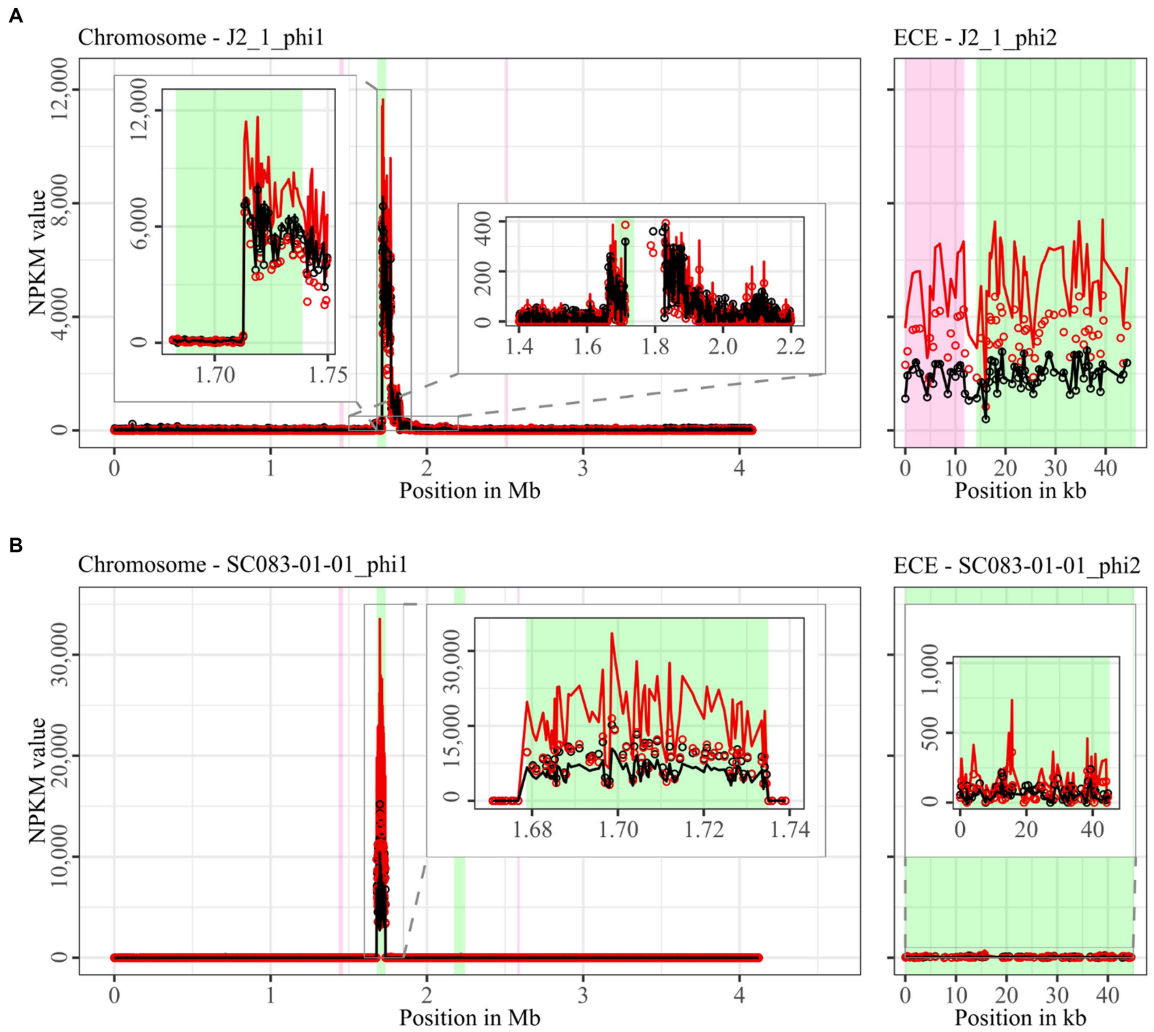
Coverage of phage sequencing reads of ST8-strains J2_1 and SC083-01-01. Sequencing reads of phage particles from spontaneous (black) and DCA-induced (red) release of strains **(A)** J2_1 and **(B)** SC083-01-01 were normalized to NPKM values (circles) with TraV (Dietrich et al., 2014), and NPKM values were additionally OD-normalized to OD_600_ = 2 (line graphs), before plotted against the chromosome (position in Mb) and ECE (position in kb) of the respective strain. Active regions were magnified for better visualization. Prophage regions predicted by PHASTEST (Wishart et al., 2023) are highlighted (intact = green, incomplete = pink).

**Figure 5:**
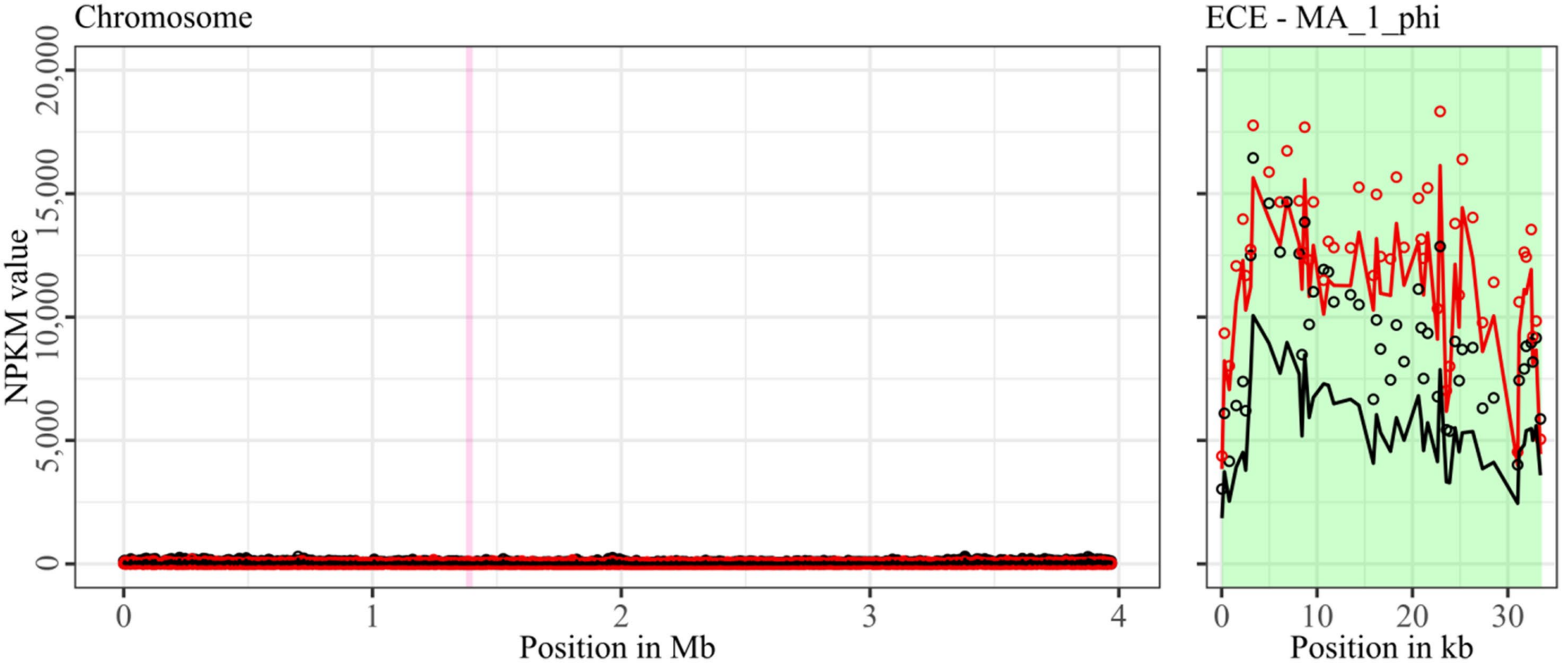
Coverage of phage sequencing reads of ST11-strain MA_1. Sequencing reads of phage particles from spontaneous (black) and DCA-induced (red) release of strain MA_1 were normalized to NPKM values (circles) with TraV (Dietrich et al., 2014), and NPKM values were additionally OD-normalized to OD_600_ = 2 (line graphs), before plotted against the chromosome (position in Mb) of MA_1. Active regions were magnified for better visualization. Prophage regions predicted by PHASTEST (Wishart et al., 2023) are highlighted (intact = green, incomplete = pink).

**Figure 6:**
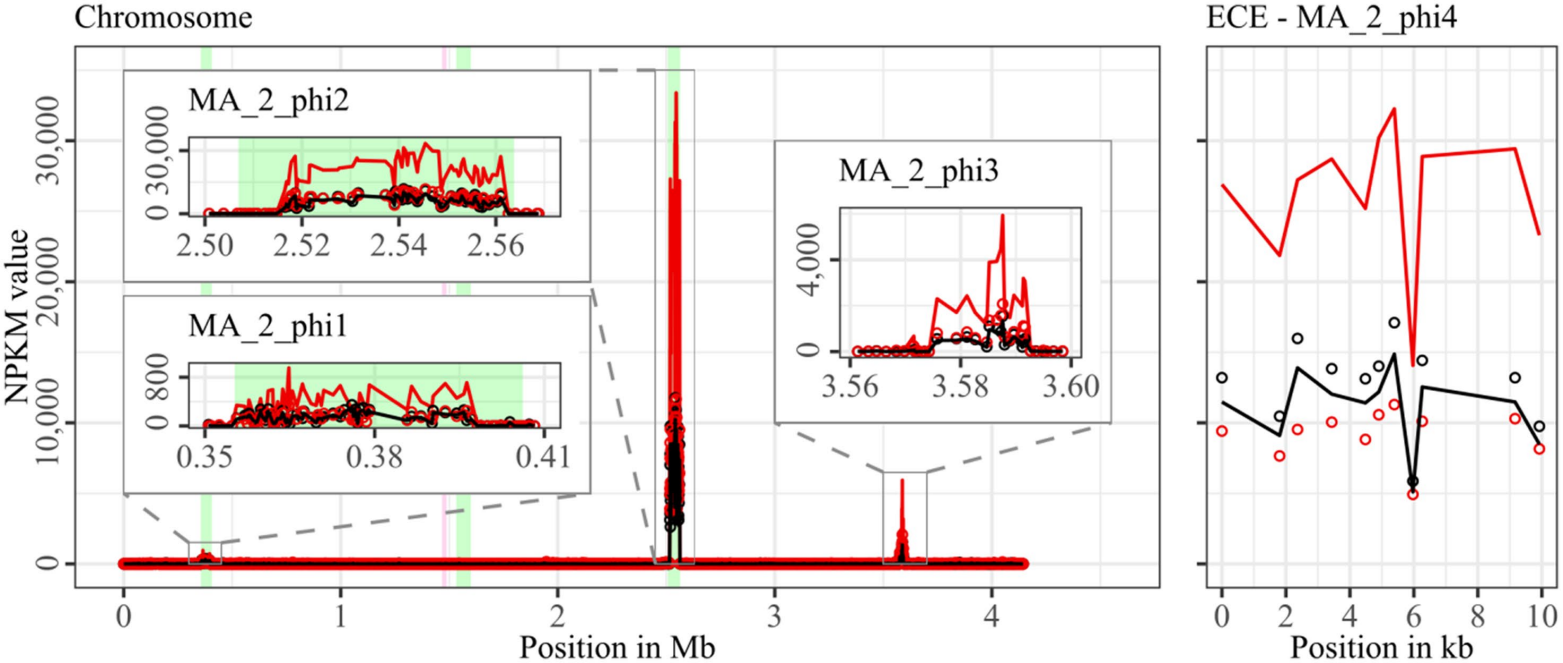
Coverage of phage sequencing reads of cryptic ST340-strain MA_2. Sequencing reads of phage particles from spontaneous (black) and DCA-induced (red) release of strain MA_2 were normalized to NPKM values (circles) with TraV (Dietrich et al., 2014), and NPKM values were additionally OD-normalized to OD_600_ = 2 (line graphs), before plotted against the chromosome (position in Mb) and ECE (position in kb) of MA_2. Active regions were magnified for better visualization. Prophage regions predicted by PHASTEST (Wishart et al., 2023) are highlighted (intact = green, incomplete = pink).

The mapping of phage DNA sequencing reads onto respective host genomes is depicted in Figures 2–6. The overall examination of the mapping results revealed distinct activity of at least one region in all strains and under both induction conditions. This demonstrated spontaneous phage activity in all strains and simultaneous activity of multiple phages within the same host. Almost all regions matched well with the predicted and intact prophage regions. All these regions are summarized in Table 2 for name assignment to facilitate the following descriptions. As apparent in Table 2, regions with positive prophage prediction accorded in size with typical genome sizes of *C. difficile* phages (Heuler et al., 2021), while those without were significantly smaller.

**Table 2:** Overview of active regions in all strains.

Strain	Location	Region name	Activity certain	Phage predicted	Size (bp)
TS3_3	Chromosome	TS3_3_phi	✓	✓	55,976
DSM 28196	Chr. region 1 Chr. region 2	DSM28196_phi1 DSM28196_phi2	✓ ✓	✓ *─*	55,976 5,484
B1_2	Chr. region 1 Chr. region 2 ECE1 ECE2	B1_2_phi1 B1_2_phi2 B1_2_phi3 B1_2_phi4	~ ✓ ✓	✓ ✓ ✓ *─*	54,334 57,163 42,358 7,624
SC084-01-01	Chromosome ECE1 ECE2	SC084-01-01_phi1 SC084-01-01_phi2 SC084-01-01_phi3	~ ~ ✓	✓ ✓ ✓	69,503 47,363 130,799
J2_1	Chromosome ECE	J2_1_phi1 J2_1_phi2	✓ ✓	✓ ✓	55,958 46,261
SC083-01-01	Chr. region 1 ECE	SC083-01-01_phi1 SC083-01-01_phi2	✓ ✓	✓ ✓	56,419 45,313
MA_1	ECE	MA_1_phi	✓	✓	33,670
MA_2	Chr. region 1 Chr. region 2 Chr. region 3 ECE	MA_2_phi1 MA_2_phi2 MA_2_phi3 MA_2_phi4	✓ ✓ ✓ ✓	✓ ✓ *─* *─*	42,327 46,234 16,820 10,144

All active regions identified based on sequencing read coverage (Figures 2–6) were renamed according to a numbered scheme (strain_phiX). Regions were validated for certain (✓) or uncertain (~) activity based on coverage signal strength. Additionally, prophage prediction with PHASTEST (Wishart et al., 2023) is included by stating positive (✓) or negative (─) prediction, and region size in bp is stated as well.

Comparison of corresponding strains showed no apparent differences in carriage or location of active phages. Correspondingly, a correlation to the clinical background of a strain was not detected. As active prophages of corresponding strains resided at corresponding genome positions, we performed an ANIm analysis on extracted sequences of all active regions to assess their similarity among each other (Supplementary Figure S1). This confirmed identical sequences of the analogous phages TS3_3_phi/DSM28196_phi1 of ST1-strains and high similarity of both phages J2_1_phi1/SC083-01-01_phi1 and J2_1_phi2/SC083-01-01_phi2 among the ST8-strains, while the other phages exhibited only little or no similarity to the others.

All spontaneously active regions (Table 2) were inspected for their activities under DCA induction. The overall NPKM transformation to an OD of 2 revealed distinctly higher signals under DCA induction in most active regions and all strains. This verified the phage-inducing effect of DCA, which varied apparently between the different regions even within the same host and thereby implied phage dependency.

The genomes of both ST1-strains carried an identical phage (Supplementary Figure S1; TS3_3_phi/ DSM28196_phi1) as determined by ANIm analysis (Supplementary Figure S1) and similar genome position. The analogous phages showed distinct spontaneous activity with approximate magnitude, but their DCA-induced activity differed substantially. Phage DSM28196_phi1 (Figure 2B) exhibited ~3.5x higher signal increase under DCA than TS3_3_phi in the non-clinical strain (Figure 2A). Since the phages were identical, the differing reactions seemed to be host-related. The genome of strain DSM 28196 possessed another active region DSM28196_phi2, which showed minor activity under both conditions and was phage-atypical by comprising only four genes and missing a prophage prediction (Figure 2B).

The genomes of ST3-strains possessed several active regions (Figure 3), which were not similar to each other (Supplementary Figure S1). In the genome of the non-clinical strain B1_2 (Figure 3A), two of the four active regions comprised the two ECEs. B1_2_phi4 on ECE2 exhibited the highest NPKM values under DCA induction. Remarkably, B1_2_phi4 is another phage-atypical but active region without corresponding prophage prediction, as previously detected for DSM28196_phi2 (Figure 2B). B1_2_phi3 on ECE1 also showed increased activity under DCA, but both spontaneous and induced activity were not particularly high. B1_2_phi2 on the chromosome showed spontaneous activity and a substantial increase in DCA-induced activity. B1_2_phi1 on the chromosome exhibited almost no signal under spontaneous conditions, which slightly increased upon DCA induction. This might indicate true DCA induction of this phage without prior spontaneous activity. In contrast to strain B1_2, the corresponding clinical strain SC084-01-01 possessed no prominently active region on the chromosome (Figure 3B). The activity could be observed within the region SC084-01-01_phi1, but NPKM values were very low under both conditions and signals did not cover the whole phage region. The abundancy of this phage was probably insufficient to capture distinct activity by the sequencing approach. Similar activity was observed for SC084-01-01_phi2 on ECE1 of SC084-01-01. SC084-01-01_phi3 on ECE2 was the only region in SC084-01-01 with prominent activity under spontaneous conditions, and the activity further increased apparently under DCA induction.

The genomes of both ST8-strains exhibited activity for their analogous chromosomal and extrachromosomal regions (Figure 4), which were similar phages according to ANIm analysis (Supplementary Figure S1) and similar genomic location. J2_1_phi2 on the ECE of strain J2_1 showed distinct spontaneous activity and a DCA-induced activity increase (Figure 4A), whereas SC083-01-01_phi1 on the ECE of SC083-01-01 was only slightly active under spontaneous conditions, and the signal increase upon DCA induction was only little. The activity of the chromosomal phages differed apparently as well. J2_1_phi1 on the J2_1 chromosome was spontaneously active and showed increased activity under DCA induction (Figure 4A). The left part of the prophage region started with minor activity, which drastically increased at the terminase genes. Strikingly, the left part of the prophage region started with minor activity, which drastically increased at the terminase genes. Further remarkable, sequencing reads mapped beyond the predicted prophage region and spread upstream (~30 kb) and downstream (~135 kb). The downstream region adjoined the phage activity with similar NPKM values that gradually decreased over the entire section. The chromosomal phage SC083-01-01_phi1 of the corresponding clinical ST8-strain SC083-01-01 did not exhibit these peculiarities (Figure 4B). It was spontaneously active, and the activity increased substantially under DCA treatment. This increase was strikingly twice as high as observed for the analogous phage J2_1_phi1 (Figure 4A), despite their similarity (Supplementary Figure S1). Such dissimilar activity increase among analogous phages was already observed in the ST1-strains (Figure 2). In both ST8- and ST1-pairs, a distinctly stronger increase upon DCA induction was connected to the clinical background of the strains. Since DCA-stress levels did not significantly differ between corresponding clinical and non-clinical strains (Figure 1) and thereby suggested similar induction levels, the question of the underlying mechanism of these diverging activities in analogous phages arose. This prompted an undefined regulation of phage induction involved in the clinical strains.

Strain MA_1 could not be compared to its corresponding clinical strain DSM 29747, but distinct spontaneous activity was visible for MA_1_phi on the ECE, which increased under DCA treatment (Figure 5).

Strain MA_2 is, to our knowledge, the first cryptic *C. difficile* strain with detailed phage examination. This strain possessed four active regions (Figure 6). MA_2_phi2 on the chromosome and MA_2_phi4 on the ECE both showed prominent activity under spontaneous conditions and a distinct activity increase upon DCA induction. Interestingly, MA_2_phi4 was another active region without phage prediction, as observed previously for DSM28196_phi2 (Figure 2B) and B1_2_phi4 (Figure 3A). This was also true for MA_2_phi3 on the chromosome, where the activity was, however, very low under spontaneous conditions. The activity of this region increased upon DCA induction. Chromosomal region MA_2_phi1 exhibited the least activity in this genome even under DCA treatment. Read mapping for MA_2_phi1 and MA_2_phi2 did not cover the entire predicted prophage regions, but sections without read coverage contained only bacterial genes and were therefore evidently mispredicted.

Almost all identified active prophage regions could be induced by the secondary bile salt DCA. Mentionable, the phage activity measured after DCA treatment might be influenced by a direct effect of DCA on the phage. A study on different bacteriophages in *Escherichia coli* investigated the effect of bile salts on the host–phage interaction and observed varying survival rates of the phages (Scanlan et al., 2017). Consequently, DCA might not only induce but also damage phages, thereby reducing the abundance of induced phage particles and the measured phage activity, respectively. The measured activity in our experiments might therefore be lower than the actual activity after induction.

DCA is not the only secondary bile salt present in the human intestines that can stress *C. difficile* cells (Sorg and Sonenshein, 2008; Thanissery et al., 2017). It might therefore be assumed that the other secondary bile salts induce prophages as well. Overall, the analysis of prophage induction and activity with all secondary bile salts—individually and combined—is advisable to further understand *C. difficile* prophage activity *in vivo.* In this regard, the corresponding toxin production of the analyzed strains would be another interesting aspect for investigations. The secondary bile salts were demonstrated to negatively affect toxin production (Thanissery et al., 2017) and function (Tam et al., 2020). In contrast, prophages can affect toxin production both negatively (Govind et al., 2009) and positively (Sekulovic et al., 2011). As such, phage induction in the presence of secondary bile salts can not only promote phage-mediated HGT and drive adaptation but also influence toxin levels upon lysogenization of other toxigenic strains, thereby further affecting strain virulence and disease severity.

Moreover, almost all ECEs were detected as active phages. Although extrachromosomal prophages have already been described in *C. difficile* strains (Garneau et al., 2018; Ramírez-Vargas et al., 2018), only a few of these have been isolated and characterized so far (Heuler et al., 2021).

### 3.3 Phage genome annotation and gene content analysis

#### 3.3.1 Phage genomes harbor virulence-relevant genes

All active regions identified via sequence read mapping (Figures 2–6; Table 2) were inspected after a new annotation with Pharokka (Bouras et al., 2023) for genes that might increase the virulence of the host (genomic information in Supplementary Data File S1). All of them exhibited characteristic phage genes in a modular organization according to the different encoded functions, as typically seen in *C. difficile* phages (Govind et al., 2006; Meessen-Pinard et al., 2012; Garneau et al., 2018). In some genomes, the protein characteristics for plasmids were found, such as genes encoding partition proteins and replication initiation factors (Filutowicz, 2009). Genes encoding plasmid-related proteins in addition to phage-typical ones are common in a certain type of MGE, so-called phage-plasmids (Pfeifer et al., 2021). These phage-plasmid features were especially recorded for extrachromosomal prophages but were also present on the chromosomally integrated prophage MA_2_phi1. Further frequently observed genes in the phage genomes encoded proteins with potential involvement in cellular metabolism and growth, such as metallo-proteases, kinases, a phosphatase, and most of all putative rhodanese-related sulfurtransferases. These genes might be advantageous for bacterial fitness, thereby indirectly contributing to host virulence. Two phage genomes (B1_2_phi1 and SC084-01-01_phi1) carried genes encoding hemolysin XhlA, an established virulence factor in other bacterial species (Thomas et al., 2021), capable of lysing mammalian erythrocytes (Cowles and Goodrich-Blair, 2004). In the opportunistic pathogen *Mannheimia haemolytica*, temperate phages were induced that encoded hemolysin XhlA and discussed to contribute to bacterial pathogenicity and transfer of this virulence factor (Niu et al., 2015). Hemolysis in *C. difficile* is not commonly known, but few studies demonstrated its hemolytic capability (Alkudmani, 2018). However, XhlA is also present in other prophage genomes as part of the lysis module (Krogh et al., 1998). Indeed, the gene encoding XhlA was found next to the lysis-relevant genes encoding holin and endolysin in phages B1_2_phi1 and SC084-01-01_phi1. Thus, the actual role of hemolysin XhlA in these phages and its potential impact on host virulence remains unclear. Phage SC084-01-01_phi1 possessed a gene encoding an ABC transporter, which might contribute to antibiotic resistance of the host (Orelle et al., 2019). Phage SC084-01-01_phi3 harbored a putative spore protease, which could influence bacterial sporulation or germination ability and, as a consequence, alter bacterial fitness (Garneau et al., 2018). Other genes conferring antibiotic resistance or encoding known virulence factors were not identified in the active phage genomes. Six phages (TS3_3_phi, DSM28196_phi1, B1_2_phi2, J2_1_phi1, SC083-01-01_phi, MA_2_phi2) carried arrays of clustered regularly interspaced short palindromic repeats (CRISPR), which is similar to other *C. difficile* phages with described CRISPRs (Hargreaves et al., 2014a; Rashid et al., 2016; Garneau et al., 2018). Temperate phages carrying CRISPR arrays increase host immunity against other invading phages (Barrangou et al., 2007). The corresponding host genomes were verified to encode Cas proteins required for CRISPR-Cas-mediated phage immunity (Koonin and Makarova, 2009). CRISPRs in prophages represent horizontally transferrable immunity against phages, which is specifically relevant in the context of phage therapy, an alternative treatment approach for bacterial infections with growing importance in view of increasing multidrug-resistances (Gutiérrez and Domingo-Calap, 2020).

#### 3.3.2 Active non-phage elements likely belong to so far undescribed HGT mechanisms in *Clostridioides difficile*

The regions without corresponding prophage prediction (Table 2) did not possess a phage-typical genome accordingly. Therefore, further gene analysis of these non-phage elements was performed based on the original genome annotation with Prokka (Seemann, 2014; genomic information in Supplementary Data File S1). No proteins involved in capsid or tail production, DNA packaging, or host lysis were present. The lack of structural proteins was striking since these DNA elements were enveloped according to the DNA isolation procedure. This indicated the involvement of unrelated particles. Even additional analysis of hypothetical proteins with InterProScan (Jones et al., 2014) and BLASTp (Johnson et al., 2008) could not identify further functions. All other proteins were assigned to functions with DNA-binding activity, such as helicases, integrases, relaxases, and transcriptional regulators. These genes are typical for phage genomes but also for MGEs such as transposons as Integrative and Conjugative or Mobilizable Elements (ICE/IME; Bellanger et al., 2014). Screening for these MGEs with ICEscreen (Lao et al., 2022) validated DSM28196_phi2 as complete IME, while MA_2_phi3 was detected as invalid ICE. Interestingly, the peculiar upstream region of J2_1_phi1 (Figure 4A) was also identified as an ICE, although it was incomplete. These integrative MGEs do not encode proteins for the production of particles that carry the respective mobile sequence (Bellanger et al., 2014). Transposons were found to hitchhike co-residing phages in several bacteria such as *Staphylococcus aureus* (Lindsay et al., 1998), *Vibrio cholerae* (Seed et al., 2013), and *Enterococcus faecalis* (Duerkop et al., 2012), enabling the phage-mediated transduction of virulence-relevant genes. This type of transduction was demonstrated once in *C. difficile* with the transfer of a conjugative transposon carrying an antibiotic resistance gene (Goh et al., 2013). Transduction can be either generalized, specialized, or lateral (Borodovich et al., 2022). They all imply the “headful” DNA packaging, in which the terminase starts DNA packaging at a bacterial homolog to the phage packaging site until the capsid is full, which consequently implies the transduced DNA to be of similar phage genome size (Borodovich et al., 2022). The transduction mechanisms differ in transduced DNA and frequency (Kleiner et al., 2020). Specialized and lateral transduction involves host DNA adjacent to the prophage, while random host DNA is packaged in generalized transduction (Kleiner et al., 2020). Generalized and specialized transduction are processes of erroneous DNA packaging, which results in low transduction frequency detectable by sequencing read coverage (Kleiner et al., 2020; Borodovich et al., 2022). In contrast, lateral transduction results in high sequencing read coverage comparable to actual phage activity, as this mechanism is considered a natural phage trait instead of a mistaken process (Kleiner et al., 2020; Borodovich et al., 2022). This trait comprises phage genome replication and simultaneous DNA packaging before excision from the chromosome, whereby a substantial amount of adjacent host DNA is packaged as well (Chiang et al., 2019; Fillol-Salom et al., 2021). All these characteristics of lateral transduction accorded with the observed sequencing read mapping downstream of phage J2_1_phi1 (Figure 4A), indicating lateral transduction of this DNA segment by phage J2_1_phi1. This observation is thereby the first description of lateral transduction in *C. difficile.* The downstream region of J2_1_phi1 (Figure 4A) did not comprise characteristic genes for MGEs. Instead, several genes encoded proteins with potential relevance for strain virulence, such as genes for ABC transporters, a multidrug efflux system ATP-binding protein, stress-related proteins, proteins involved in resistance to vancomycin and daunorubicin, and the putative virulence factor BrkB. Therefore, mobilization and transfer of this region are critical regarding the spread of antibiotic resistance or virulence-related genes. The transfer of such advantageous genes via lateral transduction involves a high transfer frequency of the DNA (Kleiner et al., 2020), which can boost the efficacy of gene dissemination in *C. difficile* and consequently promote evolutionary adaptation of the bacterial population.

The action of lateral transduction might also explain the drastic NPKM difference observed for the sequencing read mapping within the genome phage J2_1_phi1 (Figure 4A) as inaccurate excision of the phage genome after *in situ* replication leads to phage genome truncation.

Since no evidence for lateral transduction was observed for phage J2_1_phi1’s analog SC083-01-01_phi1 (Figure 4B) despite their high similarity (Supplementary Figure S1), the question about underlying differences arose. Direct phage genome comparison revealed diverging excisionases and integrases as well as four additional amino acids in the large terminase protein of J2_1_phi1. All these proteins perform activities destined for lateral transduction (Borodovich et al., 2022).

The extrachromosomal non-phage elements were significantly smaller than the co-existing phages (Table 2), which are in contrast with the headful packaging mechanism required in transduction. This indicated a form of DNA-protecting particle other than phages, such as gene transfer agents (GTA). These phage-like particles carry DNA between 4 and 14 kb (Lang et al., 2012), which is similar to the sizes of the detected non-phage elements (Table 2). However, GTAs package bacterial DNA randomly (Lang et al., 2012), which does not fit the detected distinct activity of specific regions, making GTAs unlikely as a mode of action. Extracellular vesicles are known in various bacteria and described to carry and transfer genetic content, e.g., plasmids, between cells (Fulsundar et al., 2014; Bitto et al., 2017; Tran and Boedicker, 2017). This type of alternative HGT is not well characterized so far but was demonstrated to allow interspecies gene transfer (Fulsundar et al., 2014), which underlines the importance of vesicle-mediated DNA exchange. Noteworthy, vesicle-driven HGT in *C. difficile* has not been described so far.

The envelopment of diverse MGEs could imply a more effective transfer of these DNA elements since the enveloped DNA is protected from degradation outside of the bacterial cell. This would promote the evolutionary adaptation of *C. difficile* via the spread of genes advantageous for bacterial fitness or virulence, e.g., by conferring resistance against antimicrobial substances such as antibiotics (Goh et al., 2013) or by encoding virulence-related proteins (as, for example, found in the laterally transduced chromosomal region downstream of J2_1_phi1). Many genes present in the enveloped MGEs remain of unknown function, making their potential influence on the strain’s fitness or virulence unclear. However, the data revealed the activity of these MGEs in about half of the analyzed strains and showed the considerable prevalence of these enveloped, mobile DNA elements in *C. difficile*. This indicated a significant contribution of those mechanisms in DNA transfer between *C. difficile* organisms that should be further investigated in the context of *C. difficile* adaptation and evolution. In this regard, HGT of virulence-related genes via these DNA transfer modes can be supported by close cell contact in biofilm structures that can be triggered by secondary bile salts (Dubois et al., 2019) or phage activity (Nanda et al., 2015). Moreover, phage-mediated cell lysis likely promotes the release of these various DNA transfer agents (Nanda et al., 2015), which partially showed higher activity under DCA treatment (Figures 3A, 6). A complex and dynamic interplay between the effect of DCA (or secondary bile salts in general) and the corresponding activity of phages and the other MGEs on the evolutionary adaptation of *C. difficile* can be assumed.

### 3.4 Classification of the active phages

#### 3.4.1 Terminase-based determination of the phage DNA-packaging strategy

**Figure 7:**
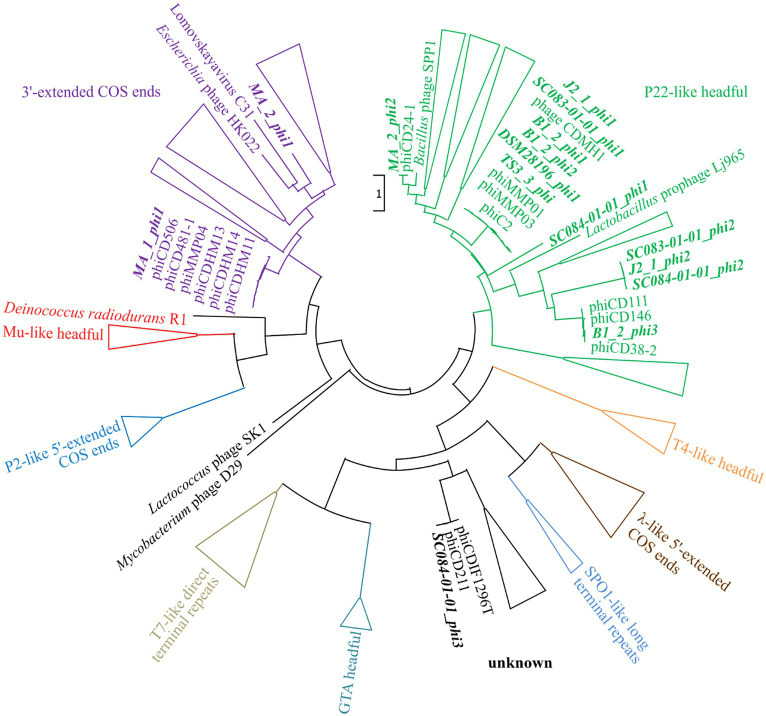
Maximum-likelihood phylogenetic tree of large terminase protein sequences. The large terminase of the active phages (highlighted in bold and italic) were aligned on the protein level to the reference sequences from Rashid et al. (2016). The branches of the different DNA-packaging strategies were colored according to Rashid et al. (2016), and the branches were collapsed for better visualization if none of our phages was included.

The assessment of the phage DNA-packaging mechanisms was performed to validate the above-hypothesized transduction events. The large terminase subunit was analyzed via protein sequence alignment and phylogenetic tree construction referring to Rashid et al. (2016). This assigned the phages to different phage DNA-packaging mechanisms (Figure 7). All our phages were assigned to clusters comprising other *C. difficile* phages, which predominantly represented the P22-like headful packaging mechanism, followed by the 3′-extended COS ends and an unknown strategy. Consequently, most of the phages were predicted to utilize the headful packaging mechanism and would, thus, be indeed capable of transducing host DNA. The mechanism “P22-like headful” originates from the packaging strategy employed by phage P22 of *Salmonella enterica*. Phage P22 was originally described to perform generalized transduction (Ebel-Tsipis et al., 1972), but recent evidence demonstrated also specialized as well as lateral transduction activity (Fillol-Salom et al., 2021). These terminase analysis results supported the assumption of lateral transduction of the phage J2_1_phi1 downstream region.

#### 3.4.2 Nucleotide BLAST analyses assess phage prevalence and novelty

A nucleotide BLAST analysis (Johnson et al., 2008) was performed on all active regions in stated Table 2 to check for similar phages and elements and to assess prevalence among *C. difficile* strains. The results are available in Supplementary Data File S2 and summarized in Table 3. Most of the phages matched against *C. difficile* phages with query coverages between 4 and 90% and percent identities between 86.26 and 99.86%. This confirmed that our phages are indeed similar to known phages but still represent novel types, which underlines the contribution of this study to the general knowledge of *C. difficile* phages. Furthermore, chromosomal phages also matched against a multitude of *C. difficile* chromosomes, while the extrachromosomal phages often corresponded to *C. difficile* plasmids and few chromosomes. This demonstrated the prevalence of the identified phages in other *C. difficile* strains. The non-phage elements B1_2_phi4 and MA_2_phi4 yielded no significant BLAST hit against a phage but matched against *C. difficile* plasmids. Both matching plasmids belong to classes of *C. difficile* plasmids with similar organization that are present in diverse *C. difficile* strains (Smits et al., 2018; Roseboom et al., 2023). All these plasmids might therefore likewise be inducible and particle-protected, which implies a different mechanism of HGT than currently assumed.

**Table 3:** Nucleotide BLAST results of the identified active regions.

	First best *C. difficile*-phage BLAST hit				
Phage	Entry	Query cover %	Percent identity %	*C. difficile* matches preceding/succeeding	Family
TS3_3_phi	CD2301	36	91.64	98 chromosomes / 32 chromosomes/assemblies 5 phages	M (Whittle et al., 2022)
DSM28196_phi1	phiC2	36	91.64	98 chromosomes / 32 chromosomes/assemblies 5 phages	M (Goh et al., 2007)
DSM28196_phi2	─	< 79	< 92.44	Only chromosomes	─
B1_2_phi1	CDMH1	60	88.33	35 chromosomes / 102 chromosomes 13 phages	M (Hargreaves et al., 2014b)
B1_2_phi2	phiC2	43	97.80	80 chromosomes / 55 chromosomes 3 phages	M (Goh et al., 2007)
B1_2_phi3	phiCD111	84	95.62	0 / 3 chromosomes 9 plasmids 16 phages	S (Sekulovic et al., 2014)
B1_2_phi4	plasmid pJMR5-4^a^	9	86.26	0 / 2 chromosomes 1 plasmid	─
SC084-01-01_phi1	phiCD418	54	92.42	29 chromosomes / 137 chromosomes 8 phages	M (Whittle et al., 2022)
SC084-01-01_phi2	HGP05	45	93.09	20 plasmids, 2 chromosomes / 145 chromosomes 14 phages	─
SC084-01-01_phi3	phiCD211	89	99.86	0 / 10 plasmids 6 phages	S (Garneau et al., 2018)
J2_1_phi1	phiC2	37	92.19	112 chromosomes / 27 chromosomes 3 phages	M (Goh et al., 2007)
J2_1_phi2	HGP05	46	91.63	20 plasmids, 2 chromosomes / 93 chromosomes 8 phages	─
SC083-01-01_phi1	phiC2	37	92.19	98 chromosomes / 43 chromosomes 4 phages	M (Goh et al., 2007)
SC083-01-01_phi2	HGP05	47	91.64	20 plasmids, 2 chromosomes / 140 chromosomes 8 phages	─
MA_1_phi	phiCD506	90	99.60	2 plasmids / 172 chromosomes 27 plasmids 20 phages	M (Sekulovic et al., 2014)
MA_2_phi1	phiCD24-1	4	88.74	17 chromosomes or genome assemblies / 0	S (Fortier and Moineau, 2007)
MA_2_phi2	phiCDKH01	74	94.21	1 chromosome / 89 chromosomes 1 phage	S (Hinc et al., 2021)
MA_2_phi3	─	< 44	< 97.72	Only chromosomes	─
MA_2_phi4	plasmid pCD-WTSI1^a^	76	91.95	0 / 25 plasmids	─

a Since ECEs B1_2_phi4 and MA_2_phi4 were identified to be no phage, BLAST results were checked for *C. difficile* plasmids instead.

Results were summarized regarding the first phage-related *C. difficile* BLAST hit by stating its description, query coverage, percent identity, and a number of preceding and succeeding chromosome/plasmid entries. The few hits of metagenome-assembled phages were not listed. If available, information on the phage family of the respective hit is indicated (M, *Myoviridae*; S, *Siphoviridae*).

#### 3.4.3 Genome-based phage assignment to *Myoviridae* and *Siphoviridae*

Finally, we classified our phages morphologically. All known *C. difficile* phages so far belong to the *Caudovirales* family of *Myoviridae* or *Siphoviridae*, which are distinguished by tail appearance (Heuler et al., 2021). Genome inspection for the presence of baseplate protein characteristics for *Myoviridae* and the length of the tail length tape measure protein as indicator for *Siphoviridae* could assign eleven phages to *Myoviridae* and four phages to *Siphoviridae* (Supplementary Table S2).

## 4 Conclusion

We aimed to investigate prophage activity in different clinical and non-clinical *C. difficile* strains and unravel the potential relationships between phage activity and the clinical background of the strain. Our investigations did not find specific connections to the clinical background, although we observed stronger DCA-related activity with clinical background for phages that were similar between clinical and non-clinical strains. We further revealed several interesting findings with relevance for future *C. difficile* phage research. We identified and characterized several active prophages in various *C. difficile* strains with a sequencing-based approach. This sensitive approach allowed the detection of multiple co-existing prophages with diverse activity. Most of these phages were distinctly active without specific induction, but they showed increased activity when induced with the secondary bile salt DCA. This proved that spontaneous activity is common in *C. difficile* prophages and that the natural stressor DCA triggers prophage induction. These findings are crucial for investigating *C. difficile* biology since secondary bile salts and phage activity evidently affect *C. difficile* fitness and virulence. Both are known to influence toxin production or promote the exchange of clinically relevant genes by triggering biofilm structures or enabling HGT. We also found genes with potential connection to virulence in some phage genomes. In this context, research on actual *in vivo* phage mobility should increasingly resemble *C. difficile*’s natural habitat. The sequencing approach further revealed active regions without phage identity. Based on genomic examinations, these regions were identified as another form of MGE, in most cases possibly integrative elements. These elements apparently participated in a strategy of mobilization that involves some kind of DNA envelopment, which pointed to phage-mediated DNA transduction, GTAs, or bacterial vesicles. This phenomenon was observed in several of the analyzed strains, which indicated that mobilization of enveloped DNA other than phage lysogeny might be a frequent mechanism in *C. difficile.* Since only one example of transduction in *C. difficile* is known so far, these mechanisms of DNA transfer via envelopment should be further investigated. One of these observations was likely the result of phage-mediated lateral transduction, thereby enabling the inter-cellular transfer of large chromosomal DNA segments. This specific type of transduction in *C. difficile* has not been described so far and opens up a new perspective on *C. difficile* phage research and HGT. Moreover, it can be assumed that the analyzed and observed aspects of DCA treatment and the associated activity of phages and enveloped MGEs interact in a complex dynamic that affects HGT and evolutionary adaptation in *C. difficile in vivo*, thereby encouraging further research.

## Data availability statement

The datasets presented in this study can be found in online repositories. The names of the repository/repositories and accession number(s) can be found in the article/Supplementary Table S3.

## Author contributions

MS: Conceptualization, Data curation, Formal analysis, Investigation, Methodology, Validation, Visualization, Writing – original draft. RD: Conceptualization, Funding acquisition, Supervision, Writing – review & editing. AP: Conceptualization, Project administration, Supervision, Validation, Writing – review & editing.

## Footnotes

^1^https://inkscape.org/de/
