# Supplementary material for: Novel insights into phage biology of the pathogen Clostridioides difficile based on the active virome
Source: Front Microbiol. 2024 Mar 21;15:1374708. doi: 10.3389/fmicb.2024.1374708 (PMC10993401; doi:10.3389/fmicb.2024.1374708)
Supplement: Supplementary file 3 [file Image_1.pdf]

Supplementary Material

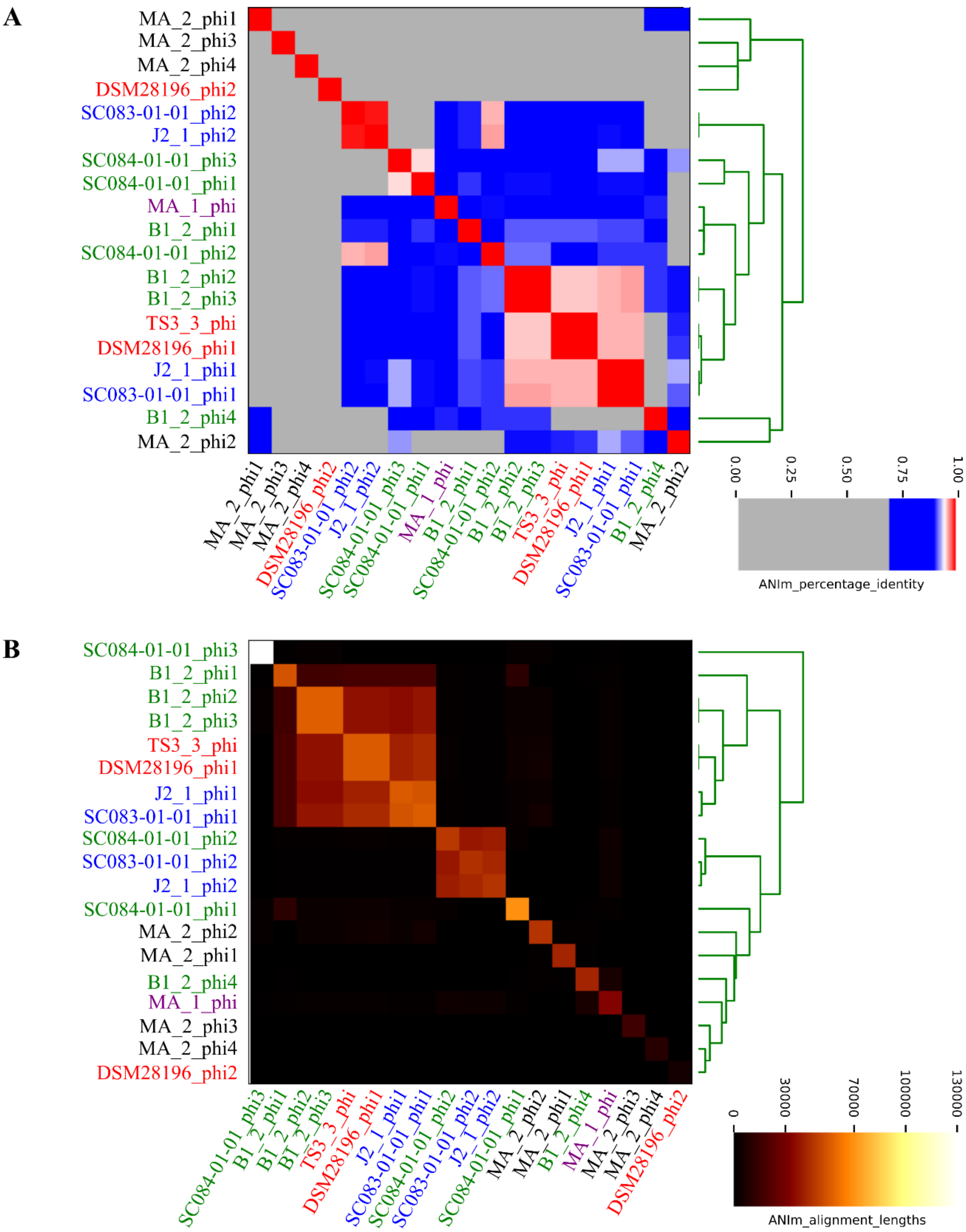

Figure S1. ANIm analysis of the active prophage regions. Heatmaps depict the **(A)** ANI values and **(B)** alignment lengths among the various active regions. The active regions are color-coded according to their ST: red = ST1, green = ST3, blue = ST8, purple = ST11, black= ST340.
