# Supplementary material for: Novel insights into phage biology of the pathogen Clostridioides difficile based on the active virome
Source: Front Microbiol. 2024 Mar 21;15:1374708. doi: 10.3389/fmicb.2024.1374708 (PMC10993401; doi:10.3389/fmicb.2024.1374708)
Supplement: Supplementary file 4 [file Table_1.docx]

Supplementary Material

Table S1. Detailed results of prophage prediction. More detailed PHASTEST (Wishart et al. 2023) results of the analyzed strains on predicted regions and completeness score.

| Strain - replicon | Predicted region | Completeness |
| --- | --- | --- |
| *C. difficile* TS3_3 | 1,437,930 – 1,465,233  1,680,766 – 1,736,741 | incomplete  intact |
| *C. difficile* B1_2  Chromosome  ECE 1  ECE 2 | 1,035,372 – 1,089,705  1,467,664 – 1,488,405  1,705,680 – 1,762,842  2,540,996 – 2,554,708  19 – 41,921  none | intact  incomplete  intact  incomplete  intact |
| *C. difficile* J2_1  Chromosome  ECE | 1,439,999 – 1,467,302  1,682,838 – 1,738,813  2,503,885 – 2,518,076  1 – 11,796  14,174 – 46,002 | incomplete  intact  incomplete  incomplete  intact |
| *C. difficile* MA_1  Chromosome  ECE | 1,375,651 – 1,402,933  28 – 33,560 | incomplete  intact |
| *C. difficile* MA_2  Chromosome  ECE | 355,247 – 406,180  1,466,266 – 1,487,446  1,532,128 – 1,599,211  2,506,900 – 2,563,763  none | intact  incomplete  intact  intact |
| *C. difficile* DSM 28196 | 1,439,999 – 1,467,302  1,682,838 – 1,738,813  2,503,885 – 2,518,076 | incomplete  intact  incomplete |
| *C. difficile* SC084-01-01  Chromosome  ECE 1  ECE 2 | 1,151,245 – 1,220,747  1,357,429 – 1,392,299  1,521,667 – 1,542,408  2,537,975 – 2,551,687  136 – 16,524  19,825 – 46,846  736 – 130,763 | intact  intact  incomplete  incomplete  incomplete  intact  intact |
| *C. difficile* SC083-01-01  Chromosome  ECE | 1,434,660 – 1,462,107  1,678,611 – 1,735,029  2,173,498 – 2,243,863  2,578,831 – 2,592,871  58 – 45,180 | incomplete  intact  intact  incomplete  intact |
| *C. difficile* DSM 29747 | 1,375,725 – 1,403,007 | incomplete |

**References**

Wishart, David S, Scott Han, Sukanta Saha, Eponine Oler, Harrison Peters, Jason R. Grant, Paul Stothard, and Vasuk Gautam. 2023. “PHASTEST : Faster than PHASTER, Better than PHAST.” *Nucleic Acids Research* gkad382: 1–8.
