# Supplementary material for: Novel insights into phage biology of the pathogen Clostridioides difficile based on the active virome
Source: Front Microbiol. 2024 Mar 21;15:1374708. doi: 10.3389/fmicb.2024.1374708 (PMC10993401; doi:10.3389/fmicb.2024.1374708)
Supplement: Supplementary file 5 [file Table_2.docx]

Supplementary Material

**Table S2. Genome-based prediction of morphological families.** Sequence length of the tail tape measure protein and presence of baseplate proteins were used to predict belonging of the phages to the family of *Myo-* (M) or *Siphoviridae* (S).

| Phage | Tail length tape measure protein length (aa) | Presence Baseplate | Family prediction |
| --- | --- | --- | --- |
| TS3_3_phi | 770 | + | M |
| DSM28196_phi1  DSM28196_phi2 | 770  ─ | +  ─ | M  ─ |
| B1_2_phi1  B1_2_phi2  B1_2_phi3  B1_2_phi4 | 764  797  1,767  ─ | +  +  ─  ─ | M  M  S  ─ |
| SC084-01-01_phi1  SC084-01-01_phi2 SC084-01-01_phi3 | 1,416  1,130  2,000* | +  +  ─ | M  M  S |
| J2_1_phi1  J2_1_phi2 | 797  1,129 | +  + | M  M |
| SC083-01-01_phi1  SC083-01-01_phi2 | 797  1,129 | +  + | M  M |
| MA_1_phi | 584 | + | M |
| MA_2_phi1  MA_2_phi2  MA_2_phi3  MA_2_phi4 | 2,226  1,839  ─  ─ | ─  ─  ─  ─ | S  S  ─  ─ |

*SC084-01-01_phi1 possessed two putative tail length tape measure proteins in close proximity. This is similar to the recent entry of phiCD211 (NC_029048.2), whereas the smaller protein is annotated as minor tail protein in the old entry LN681537.2.
