# Supplementary material for: Novel insights into phage biology of the pathogen Clostridioides difficile based on the active virome
Source: Front Microbiol. 2024 Mar 21;15:1374708. doi: 10.3389/fmicb.2024.1374708 (PMC10993401; doi:10.3389/fmicb.2024.1374708)
Supplement: Supplementary file 6 [file Table_3.docx]

Supplementary Material

**Table S3. Accession numbers of sequencing raw data, available at the Sequencing Read Archive (SRA;** [**https://www.ncbi.nlm.nih.gov/sra/**](https://www.ncbi.nlm.nih.gov/sra/)**).**

| Strain | Spontaneous | DCA-induced |
| --- | --- | --- |
| TS3_3 | SRR26060497 | SRR26060498 |
| DSM 28196 | SRR26060430 | SRR26060431 |
| B1_2 | SRR26060462 | SRR26060463 |
| SC084-01-01 | SRR26064407 | SRR26064408 |
| J2_1 | SRR26060485 | SRR26060486 |
| SC083-01-01 | SRR26060487 | SRR26060488 |
| MA_1 | SRR26060489 | SRR26060490 |
| MA_2 | SRR26064320 | SRR26064321 |
